# Impact of Imiglucerase Supply Shortage on Clinical and Laboratory Parameters in Norrbottnian Patients with Gaucher Disease Type 3

**DOI:** 10.1007/s00005-014-0308-8

**Published:** 2014-09-10

**Authors:** Maciej Machaczka, Cecilia Kämpe Björkvall, Joanna Wieremiejczyk, Martin Paucar Arce, Kristina Myhr-Eriksson, Monika Klimkowska, Hans Hägglund, Per Svenningsson

**Affiliations:** 1 Medical Faculty, University of Rzeszow, 35-959 Rzeszow, Poland; 2 Hematology Center Karolinska and Department of Medicine at Huddinge, Karolinska Institutet, Karolinska University Hospital Huddinge, M54, Stockholm, Sweden; 3 Department of Medicine, Sunderby Regional Hospital of Norrbotten County, Luleå, Sweden; 4 Department of Neurology, Karolinska University Hospital Huddinge, Stockholm, Sweden; 5 Department of Clinical Neuroscience, Karolinska Institutet, Stockholm, Sweden; 6 Department of Clinical Pathology and Cytology, Karolinska University Hospital Huddinge, Stockholm, Sweden

**Keywords:** Cerezyme™, Enzyme replacement therapy, Gaucher disease, Imiglucerase, Norrbottnian type, Shortage, Supply

## Abstract

A viral contamination of the production plant producing imiglucerase (Cerezyme™) resulted in an unpredicted worldwide shortage of global supplies during 2009–2010. The aim of the study was to describe the effects of dose reduction of enzyme replacement therapy (ERT) in adults with Norrbottnian form of Gaucher disease type 3 (N-GD3). There were ten adults with N-GD3 treated with imiglucerase in the county of Norrbotten in June 2009. Analyzed variables included plasma chitotriosidase activity and concentration of CCL18/PARC, whole blood hemoglobin concentration (Hb) and platelet count (PLT), as well as patients’ body weight, subjective complaints and health status measured by the EuroQoL-5D questionnaire. The median duration of ERT shortage lasted for 14 months (10–20 months). The median percentage reduction of imiglucerase dose was 36 % (26–59 %). Hb decreased in four patients, PLT decreased in three patients, chitotriosidase increased in three patients (max. +22 % of baseline), and CCL18/PARC increased in six patients (+14 % to +57 %). The body weight was moderately decreased in one patient. No new bone events were noted. Self-assessment of individual patient’s health status was stable in all but one patient. Our results suggest that moderate reduction of ERT dosage lasting for relatively short period of time can lead to worsening in biomarkers of adults with N-GD3. However, this worsening is infrequently translated to clinical worsening of patients. It is possible that CCL18/PARC has a higher sensitivity than chitotriosidase in monitoring of ERT dosing in GD3.

## Introduction

Gaucher disease is a pan-ethnic lysosomal storage disorder which belongs to the so-called ultra rare diseases affecting less than 20 individuals/million inhabitants (or <1 persons/50,000 inhabitants). It is caused by a deficient activity of the lysosomal enzyme glucocerebrosidase (GBA), resulting from autosomal recessive mutations in the *GBA1* gene (1q21) (Machaczka 2013; Zimran 2011). Decreased GBA activity leads to accumulation of glucosylceramide in monocytes-macrophages throughout the body, and in severe disease forms leads to neurodegenerative changes in the central nervous system (Erikson et al. 1997; Machaczka et al. 2011).

There are three major clinical subtypes of Gaucher disease, distinguished by the presence of neurologic symptoms and the dynamics of their development: an acute neuronopathic form (type 2), a chronic neuronopathic form (type 3), and a non-neuronopathic form (type 1) (Machaczka 2013; Zimran 2011). Thrombocytopenia, anemia, splenomegaly, hepatomegaly, and bone manifestations are the most typical signs of Gaucher disease type 1 (GD1), which is the most prevalent form of Gaucher disease (Machaczka et al. 2012b; Zimran 2011). Besides the aforementioned symptoms, the presence of central nervous system disease is a hallmark of Gaucher disease type 2 and 3 (GD2 and GD3) (Erikson et al. 1997).

Although the neuronopathic forms are the rarest variants of Gaucher disease, an endemic cohort of Swedish patients with chronic neuronopathic Gaucher disease lives in Northern Sweden in the county of Norrbotten, an area of approximately 100,000 square km, which is inhabited by approximately 250,000 people. This unique form of Gaucher disease, belonging to the subtype 3B of Gaucher disease, is called the Norrbottnian form of Gaucher disease (N-GD3) (Dreborg et al. 1980). In Sweden, N-GD3 consists of approximately 40 % of all known cases of Gaucher disease.

Since the early 1990s, enzyme replacement therapy (ERT) with macrophage-targeted recombinant GBA is the standard of care in moderate to severe Gaucher disease (Erikson et al. 2006; Machaczka et al. 2012a; Zimran 2011). Until 2009, imiglucerase (Cerezyme™, Genzyme Corporation, Cambridge, MA, USA) was the only ERT available for treatment of patients with Gaucher disease in the European Union (Hollak et al. 2010). In June 2009, the Genzyme Corporation announced a vesivirus 2117 contamination of the production plant, which led to a worldwide supply shortage of Cerezyme™ over 1 year. This resulted in an unintentional discontinuation of treatment for many hundreds of patients with milder forms of Gaucher disease (GD1) and a significant dose reduction for those with moderate and severe forms of Gaucher disease (GD1 and GD3) (Deroma et al. 2013; Giraldo et al. 2011; Goldblatt et al. 2011; Zimran et al. 2011).

The aim of this retrospective analysis is to describe the effects of the imiglucerase shortage in adults with N-GD3 to increase the knowledge of therapy effects on type 3 Gaucher disease. To our knowledge, this is the only study describing what happened to patients with GD3 during the ERT shortage.

## Materials and Methods

### Patients

There were 15 patients with N-GD3 in the county of Norrbotten in June 2009. Twelve of them were adults aged 21–58 years and followed at the Department of Medicine, Sunderby Regional Hospital of Norrbotten County in Luleå, Sweden. Ten of the 12 patients (four females and six males) were treated with imiglucerase in June 2009. Two of the twelve patients successfully underwent matched-related allogeneic bone marrow transplantation as children and thus do not require ERT (Ringdén et al. 1995). One man died in November 2009 due to esophageal cancer. The remaining nine patients were suitable for this long-term impact study, analyzing changes in clinical and laboratory parameters of Gaucher disease due to the unintended dose reduction of imiglucerase (Cerezyme™). Patient characteristics are presented in Table 1, including a retrospective assessment using the modified severity scoring tool for neuronopathic Gaucher disease according to Davies et al. (2011).

**Table 1:** Characteristics of adults with the Norrbottnian form of Gaucher disease in June 2009

Characteristic	Result
Total number of patients	12
Men	6 (50 %)
Women	6 (50 %)
Median age (range) (years)	42 (21–58)
Number of splenectomized patients	11 (92 %)
Partial splenectomy	2/11
Median age at the time of splenectomy (range) (years)	7 (1–20)
Number of patients with bone disease	12/12 (100 %)
Number of patients with diagnosed epilepsy	7/12 (58 %)
Number of patients on ERT in June 2009	10/12 (83 %)
Median duration of ERT in June 2009 (range) (years)	17 (8–17)
mSST of patients included in the study (*n* = 9)	
mean mSST	10
median mSTT (range)	9 (1–23.5)

*ERT* enzyme replacement therapy, *mSST* modified severity scoring tool

The patient files were reviewed for the collection of relevant clinical data. Patients provided their informed consent. Analyzed variables included results of clinical examination and Gaucher disease biomarkers: plasma chitotriosidase activity (control range <40 nkat/L) and plasma concentration of chemokine (C–C motif) ligand 18/pulmonary and activation-regulated chemokine (CCL18/PARC; control range <100 µg/L). Furthermore, values of the whole blood hemoglobin concentration (Hb; control range: 117–153 g/L), and whole blood platelet count (PLT; control range 165–387 × 10^9^/L) were analyzed. Assessment of the aforementioned variables was performed at baseline (the last examination before imiglucerase supply shortage) and at follow-up until the ERT shortage was resolved.

Each patient’s body weight was documented at the onset of as well as during the imiglucerase supply shortage period. Unexpected events occurring while on reduced ERT doses were reported by patients to their physicians and documented in patient files. The EuroQoL-5D (EQ-5D) questionnaire, which is a generic instrument to evaluate the current general state of health, was used to assess changes in N-GD3 patient’s health status before and at the end of the imiglucerase shortage period (Sanchez-Arenas et al. 2014).

## Results

The median duration of ERT supply shortage for N-GD3 patients lasted for 14 months (range 10–20 months). Characteristics of individual patient’s ERT doses during imiglucerase supply shortage are shown in Table 2. For the entire N-GD3 group, the absolute imiglucerase dose reduction was from the median of 2,800 units/dose to the median of 1,650 units/dose given every other week. The median percentage reduction of imiglucerase dose was −36 % compared with the imiglucerase dosage before June 2009 (dose reduction was uncertain in one patient due to his ERT self-administration and presumably, some saved imiglucerase doses). After 1 year of imiglucerase shortage, two patients were switched to ERT with velaglucerase (VPRIV™, Shire HGT, Lexington, MA, USA). After the shortage period, imiglucerase was restarted in the pre-shortage dosage in all patients.

**Table 2:** Characteristics of individual patient ERT doses during the imiglucerase supply shortage

Pt	Total individual imiglucerase doses before supply shortage^a^ (U/kg/infusion)	Median imiglucerase doses during supply shortage^a^	Mean percentage reduction in imiglucerase dose as compared to baseline (%)	Duration time of ERT with reduced doses (in months)
1	2,400 (40)	1,200	−36	13
2	3,200 (65)	1,600	−57	20
3	3,000 (48)	2,000	−36	14
4	2,800 (54)	2,000	−59	10
5	2,400 (67)	1,800	−37	14
6	2,400 (28)	1,700	−36	14
7	4,000 (68)	N/A	N/A	14
8	1,600 (46)	1,600	−26	10
9	3,600 (69)	1,600	−56	16

*Pt* patients, *N/A* not applicable

^a^units/dose administered every other week

**Fig. 1:**
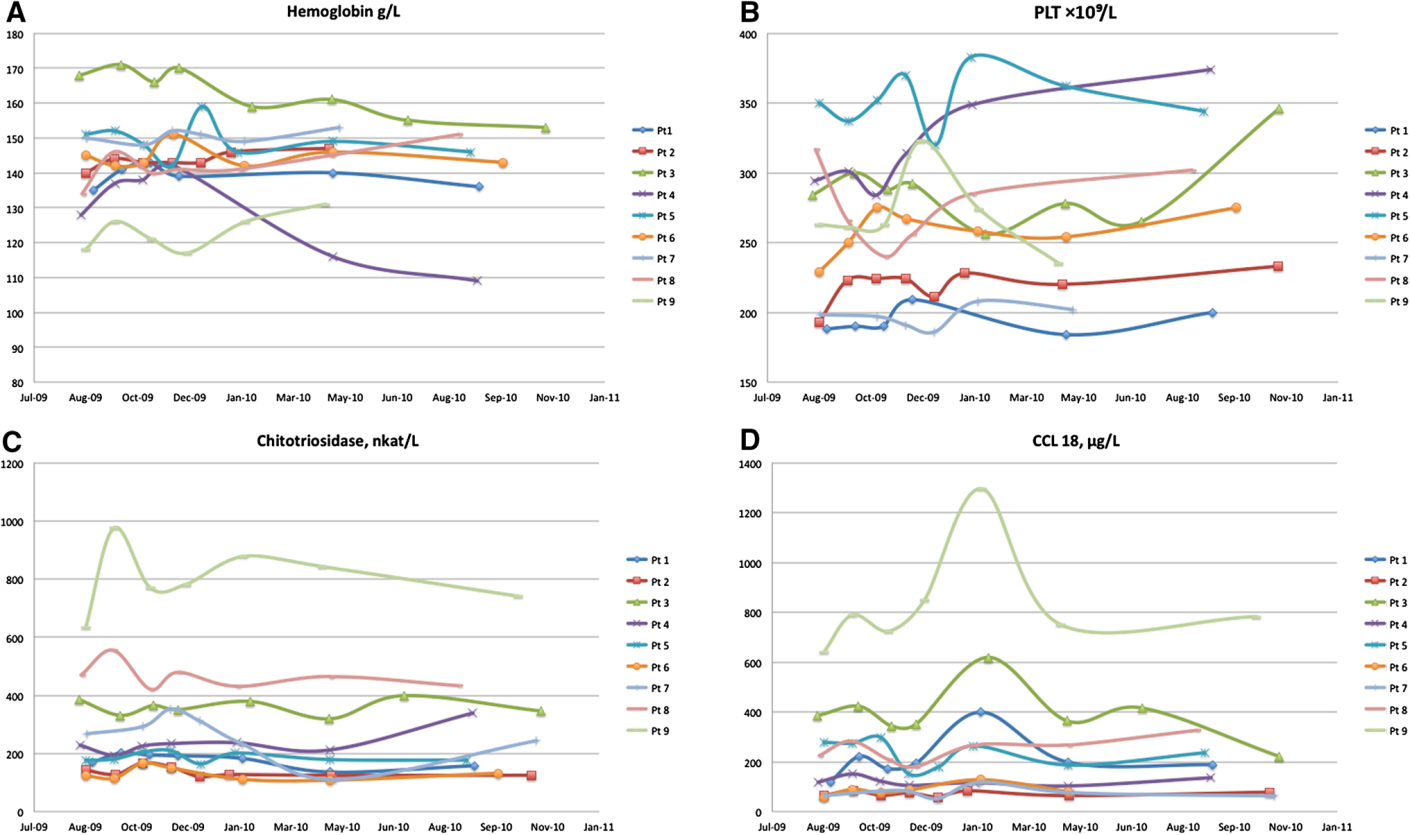
Individual patient serial **a** hemoglobin concentration, **b** platelet counts, **c** chitotriosidase activity, and **d** CCL18/PARC concentration during the ERT shortage period

Serial measurements of Hb, PLT, chitotriosidase activity, and CCL18/PARC concentration during the ERT shortage period are presented in Fig. 1a–d for each individual patient. The changes in Hb, PLT, chitotriosidase, and CCL18/PARC observed at baseline and at the end of the ERT supply shortage for each patient are summarized in Table 3.

**Table 3:** Individual patient changes in hematological parameters (Hb, PLT), biomarkers (chitotriosidase, CCL18/PARC), and body weight observed before and at the end of the ERT supply shortage

Pt	Hemoglobin		Platelet count		Chitotriosidase		CCL18/PARC		Body weight	
	A (g/L)	B (%)	A (×10^9^/L)	B (%)	A (nkat/L)	B (%)	A (μg/L)	B (%)	A (kg)	B (%)
1	135	+0.7	188	+6	171	−8	120	+57	60	+5
2	140	+3	193	+21	144	−13	64	+22	49	+2
3	168	−9	284	+22	384	−10	384	−42	62	+1
4	128	−15	294	+27	277	+22	119	+14	52	−2
5	151	−3	350	−2	175	0	278	−15	36	+4
6	145	−1.4	229	+20	123	+7	59	+36	86	+2
7	150	+2	198	+2	267	−9	63	0	59	0
8	134	+13	317	−5	471	−8	226	+44	35	+1
9	118	+11	263	−11	635	+17	640	+22	52	−9

*Pt* patients, *A* before imiglucerase shortage, *B* percentage change at the end of ERT supply shortage as compared to baseline

Hemoglobin concentration decreased in four (44 %) patients; however, only in one of them was the Hb value below the reference range (Hb 109 g/L in patient 4). Hemoglobin concentration increased in five (56 %) patients; in three of them ≤3 %. PLT decreased in three (33 %) patients; minimal changes were noted in two patients and moderate changes in one. Notably, none of the analyzed patients developed thrombocytopenia. On the other hand, PLT increased in six (67 %) patients; in four of them ≥20 %.

Both chitotriosidase activity and CCL18/PARC concentration showed fluctuations, with strongly increased levels particularly in the first 6 months of ERT supply shortage. However, further follow-up showed that at the end of the shortage period chitotriosidase activity was increased only in three (33 %) patients and observed activity changes were moderate (up to +22 % as compared to baseline). Six (67 %) patients showed stable or moderately decreased chitotriosidase activity (≤13 %). Concentration of CCL18/PARC at the end of the ERT supply shortage was increased in six (67 %) patients, and observed concentration changes were moderate to severe in all patients (from +14 % up to +57 % as compared to baseline). Three (33 %) patients presented stable or moderately decreased concentration of CCL18/PARC. In four (44 %) patients both chitotriosidase and CCL18/PARC clearly displayed the same trend in the direction of changes (Table 3).

**Fig. 2:**
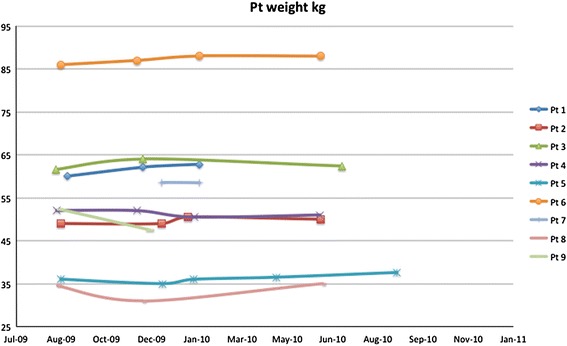
Changes in body weight of individual patients

During the imiglucerase supply shortage, body weight was minimally decreased in one patient and moderately decreased in one patient (−9 % as compared to baseline). Seven (78 %) patients showed stable or moderately increased body weight (≤5 %). Changes in body weight of individual patients are shown in Fig. 2.

No new bone events were noted for the entire group during the whole ERT supply shortage period. Interestingly, one N-GD3 patient developed epilepsy at the age of 45 years, a severe and handicapping complication of GD3. Epilepsy developed 8 months after the end of the imiglucerase shortage and 22 months after the start of the imiglucerase shortage (the patient’s mean dose reduction of imiglucerase was 36 % as compared to baseline).

**Fig. 3:**
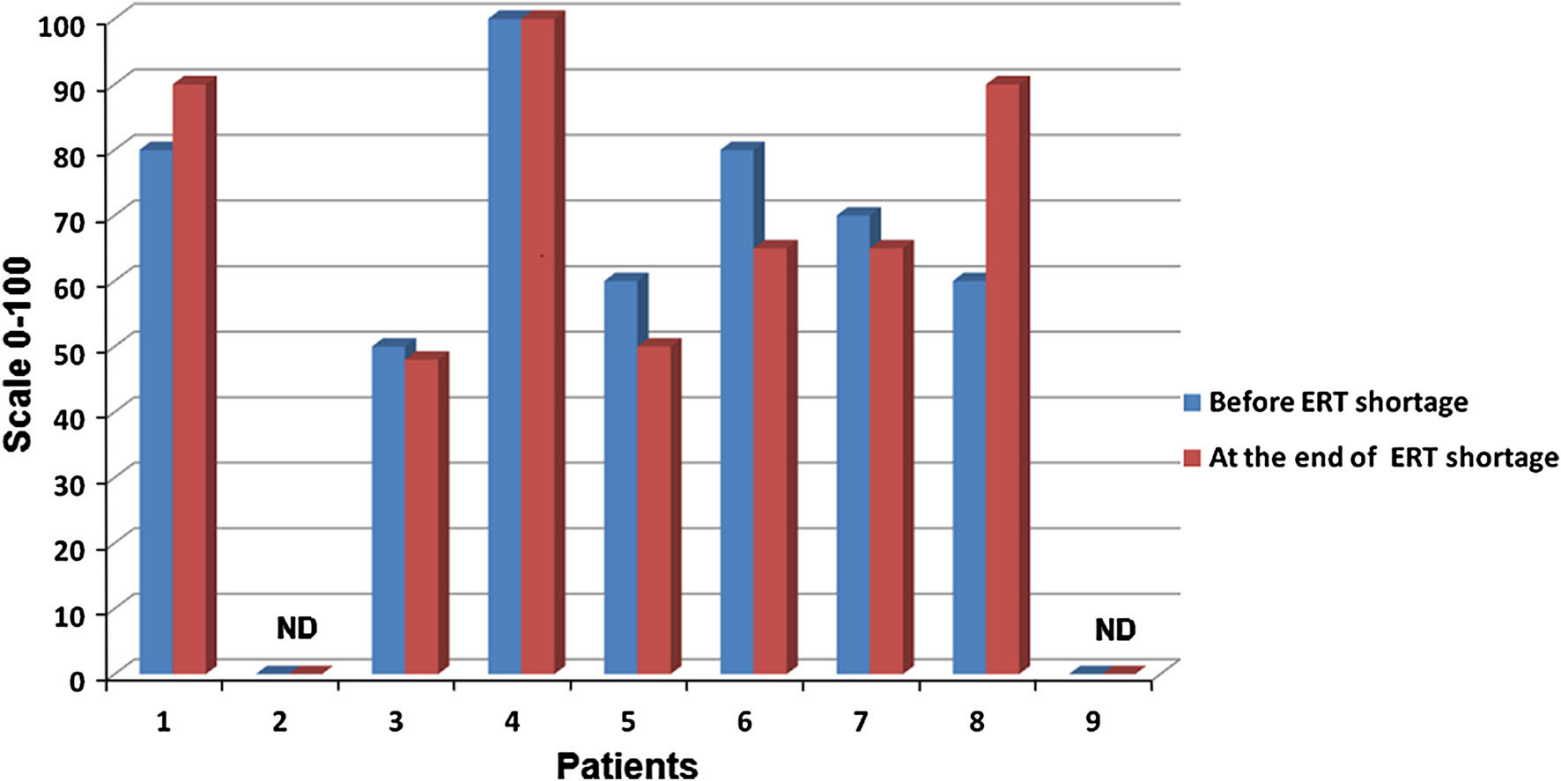
Changes in patient’s health status measured by the EQ-5D. *ND* not done

Seven (78 %) patients were able to report changes in their mobility, self care, usual activities, pain or discomfort, and anxiety or depression, during the ERT supply shortage period, as assessed by the EQ-5D questionnaire. Changes in individual patient’s health status measured by the EQ-5D are presented in Fig. 3.

## Discussion

The Norrbottnian form is a well-characterized subtype of type 3 Gaucher disease, with the first clinical symptoms occurring at the median age of 1 year. It is both an aggressive systemic disease and a slowly progressive heterogeneous neurological syndrome (Svennerholm et al. 1991). Beyond the typical hematologic and visceral symptoms of GD1, the most common signs of N-GD3 are horizontal supranuclear gaze palsy and skeletal involvement with kyphoscoliosis as a predominant skeletal manifestation (Dreborg et al. 1980). Other manifestations include convergent squint (due to abducens nerve palsy), retinal infiltrates, ataxia, mild spasticity in the legs, epilepsy (myoclonic or complex partial seizures), and a slow cognitive decline into dementia (Dreborg et al. 1980; Svennerholm et al. 1991). Genetic studies have showed that N-GD3 is caused by homozygous mutations c.1448T > C (L444P) in the *GBA1* gene (Dahl et al. 1990). Without treatment, 50 % of N-GD3 patients die before the age of 12 years (Dreborg et al. 1980).

An unpredicted shortage in the global supplies of imiglucerase in 2009 resulted in worldwide withdrawal or dose reduction of imiglucerase for the vast majority of Gaucher patients (Deroma et al. 2013; Giraldo et al. 2011; Goldblatt et al. 2011; Hollak et al. 2010; Zimran et al. 2011). According to the provided guidelines, there was a general agreement that priority should be given to the most vulnerable patients (infants, children, adolescents, pregnant women, and adults with severe Gaucher disease, including GD3) (Hollak et al. 2010). The consequences of this situation in GD1 have been described by others at approximately 6 months (Giraldo et al. 2011; Goldblatt et al. 2011; Zimran et al. 2011) and 12 months (Deroma et al. 2013) after the start of the imiglucerase supply shortage.

Although the symptomatology and clinical outcome of treated and untreated patients with GD1 and GD3 differ significantly, no published data about the impact of the forced ERT reduction in GD3 (consisting approximately 5 % of all known cases of Gaucher disease) has been available until now.

In our patients with N-GD3, Hb decreased below the reference range in only one woman. Noteworthy, she also had difficulties in keeping a normal Hb before the imiglucerase supply shortage (despite splenectomy), and was permanently treated with erythropoietin with a good Hb response. Although PLT decreased in 33 % patients, none of them developed thrombocytopenia. Until 1965, splenectomy was routinely performed as a life-saving procedure at the time of N-GD3 diagnosis or shortly thereafter to protect the patient from lethal bleeding (Dreborg et al. 1980). After splenectomy peripheral blood PLTs have increased promptly and for many years without any additional therapy. We think that generally preserved PLT and Hb during the ERT supply shortage probably depend on the fact that all but one patient of those analyzed had been splenectomized. Our results are in line with the results observed by some other authors with respect to GD1 (Deroma et al. 2013; Giraldo et al. 2011; Goldblatt et al. 2011). Two of such studies revealed that even if laboratory parameters remained stable in most GD1 patients after approximately 6 months of ERT withdrawal, their clinical status worsened (Giraldo et al. 2011; Goldblatt et al. 2011).

Analysis of Gaucher disease biomarkers in N-GD3 patients after 14 months of the imiglucerase supply shortage showed more pronounced increases in CCL18/PARC concentration than in chitotriosidase activity. We speculate that this finding suggests a higher sensitivity of CCL18/PARC than chitotriosidase in monitoring of the ERT dose in GD3. Based on these data, it may be considered that routine examination of CCL18/PARC should be introduced in centers treating GD3 which are still in favor of sole examination of chitotriosidase. Of note, a recent study by Deroma et al. (2013) disclosed a significant increase in chitotriosidase activity in non-splenectomized GD1 patients and non-significant increase in splenectomized GD1 patients 12 months after the ERT dose reduction. This is in line with our results observed among N-GD3 patients, but in contrast with the earlier study of Czartoryska et al. (2000) showing a more pronounced increase in chitotriosidase activity in splenectomized GD1 and GD3 patients approximately 3 months after ERT withdrawal. On the other hand, Giraldo et al. (2011) have found that chitotriosidase activity was more increased in GD1 patients who discontinued ERT rather than those who continued treatment but with reduced doses of ERT. In the same study, CCL18/PARC showed no significant changes during the ERT shortage period, and the authors concluded that probably this biomarker of Gaucher disease is less sensitive than chitotriosidase. The aforementioned results observed among Spanish GD1 patients are in contrast to our own results obtained in the cohort of adults with N-GD3. Nevertheless, we think that whenever possible, it is reasonable to introduce measurements of CCL18/PARC beside chitotriosidase in the follow-up of GD3 patients.

Visceral disease (splenomegaly, hepatomegaly) was not systematically followed up in our study due to the fact that vast majority of our patients were splenectomized. Moreover, many patients can not or did not want to be a subject of frequent radiological examinations. However, Zimran et al. (2011) have reported an increase in organomegaly in GD1 patients approximately 6 months after the ERT dose reduction.

Both body weight and bone disease were stable in our patients. In the group of GD1 patients, however, Giraldo et al. (2011) have found a significant increase in the incidence of bone crisis (20 %) during the period of ERT supply shortage.

Although no causal data exist, we cannot exclude that in one 45-year-old patient the reduced ERT dose was a contributing factor in the development of epilepsy 8 months after the end of imiglucerase shortage. This is due to the assumption that epilepsy in the ERT-treated N-GD3 patients, if present, usually starts at earlier age (median 24 years; range 10–27 years).

When the imiglucerase shortage occurred, many patients reported fatigue. On the other hand, they also reported being worried about a possible influence of the ERT withdrawal or dose reduction on the outcome of their disease (Deroma et al. 2013). In our cohort, a self-assessment of individual patient’s health status measured by the EQ-5D questionnaire was stable in all but one patient.

To the best of our knowledge, this study is the only published report on the impact of the imiglucerase supply shortage on clinical and laboratory parameters in GD3 in general, and in Norrbottnian GD3 in particular.

## Conclusions

The results of our retrospective analysis suggest that a moderate reduction of ERT dosage lasting for relatively short period of time in clinically stable adult patients can lead to worsening in biomarkers, clinical symptoms, and health status in adults with N-GD3. However, this worsening is infrequently translated to clinical worsening of patients. It is possible that CCL18/PARC has a higher sensitivity than chitotriosidase in monitoring of ERT dose in GD3.
